# Comparison of Comfort and Effectiveness of Total Face Mask and Oronasal Mask in Noninvasive Positive Pressure Ventilation in Patients with Acute Respiratory Failure: A Clinical Trial

**DOI:** 10.1155/2017/2048032

**Published:** 2017-02-08

**Authors:** Somayeh Sadeghi, Atefeh Fakharian, Peiman Nasri, Arda Kiani

**Affiliations:** ^1^Al-Zahra Hospital, Isfahan University of Medical Sciences, Isfahan, Iran; ^2^Chronic Respiratory Diseases Research Center, Shahid Beheshti University of Medical Sciences, Tehran, Iran; ^3^Department of Pediatric Gastroenterology and Hepatology, Emam Hosein Hospital, Isfahan University of Medical Sciences, Isfahan, Iran; ^4^Tracheal Diseases Research Center, Shahid Beheshti University of Medical Sciences, Tehran, Iran

## Abstract

*Background*. There is a growing controversy about the use of oronasal masks (ONM) or total facemask (TFM) in noninvasive positive pressure ventilation (NPPV), so we designed a trial to compare the uses of these two masks in terms of effectiveness and comfort.* Methods*. Between February and November 2014, a total of 48 patients with respiratory failure were studied. Patients were randomized to receive NPPV via ONM or TFM. Data were recorded at 60 minutes and six and 24 hours after intervention. Patient comfort was assessed using a questionnaire. Data were analyzed using* t*-test and chi-square test. Repeated measures ANOVA and Mann–Whitney* U* test were used to compare clinical and laboratory data.* Results*. There were no differences in venous blood gas (VBG) values between the two groups (*P* > 0.05). However, at six hours, TFM was much more effective in reducing the partial pressure of carbon dioxide (PCO2) (*P* = 0.04). Patient comfort and acceptance were statistically similar in both groups (*P* > 0.05). Total time of NPPV was also similar in the two groups (*P* > 0.05).* Conclusions*. TFM was superior to ONM in acute phase of respiratory failure but not once the patients were out of acute phase.

## 1. Introduction

Oronasal masks (ONM) have historically been the preferred choice in acute cases because of reduced air leak from the mouth, while nasal masks are preferred for prolonged ventilation as they do not cover the patient's mouth and provide more comfort [1, 2]. However, one-third of patients refuse both ONM and nasal masks due to air leak, face discomfort, and claustrophobia [3, 4]. There is a growing controversy about using ONM as the first choice in patients with acute respiratory failure [1, 2, 5] while total face mask (TFM) has been developed to improve patient compliance. While some authors reported better patient tolerance and reduced air leak in patients using TFM [6, 7], others have reported no difference between the two masks in terms of efficacy and outcome [7]. To date, there is no evidence supporting the clinical superiority of TFM in spite of its improved acceptance by the patients. Thus, this trial was designed to compare TFM and ONM in terms of effectiveness and comfort in patients with acute respiratory failure who were receiving noninvasive positive pressure ventilation (NPPV).

## 2. Materials and Methods

The study protocol was approved by the ethics committee of Masih Daneshvari Hospital and registered at http://www.irct.ir/ (IRCT2016051627929N1). This randomized controlled trial was conducted on 48 patients with acute respiratory failure who were referred to Masih Daneshvari Hospital from February to November of 2014. The diagnosis of acute respiratory failure was made based on medical history and thorough clinical examination. Patients were enrolled if they showed at least one of the following signs: tachypnea (respiratory rate ≥ 24), use of accessory muscles of respiration (defined as contraction of sternocleidomastoid and intercostal, suprasternal, or supraclavicular retractions during inhalation), paradoxical respiration, pH < 7.35 with no metabolic component, and finally PaCO_2_ > 45 mmHg while breathing room air. Patients with fluctuating mental status, unstable coronary artery disease, arrhythmias with unstable blood pressure (SBP ≤ 90 mm Hg or > 40 mmHg drop in systolic blood pressure), generalized skin lesions, facial trauma, upper airway obstruction, and recent surgery on upper airways, stomach, or esophagus and those who required emergent intubation were excluded. Six patients had problems with using the assigned masks due to previous unpleasant experiences or difficulties in adapting the mask to their face and consequently were excluded after randomization.

### 2.1. NPPV Technique

Patients with acute respiratory failure who were referred to Masih Daneshvari Hospital were divided into two groups, using simple randomization with a table of random numbers. Patients in the first group received NPPV through ONM (Respironics Amara® full face mask: Philips Respironics, United States) while the patients in the second group received NPPV via TFM (Respironics FitLife® mask SE: Philip Respironics, United States).

NPPV was delivered by trained personnel in Masih Daneshvari Hospital (ward and emergency room) under the supervision of pulmonary and critical care fellows. The patients played no role in selection of the mask and the statistician was blinded to the group allocation of patients. Ventilator (ResMed®Stellar 150: by Germany Inc. Fraunhoferstr, Germany) was used for all patients. NPPV parameters were set based on prior reports for 24 hours continuously. We started with inspiratory positive airway pressure (IPAP) of 10 and expiratory positive airway pressure (EPAP) of 4 cm H_2_O. Based on the severity of disease and physical symptoms, IPAP and if necessary EPAP were gradually increased within the patient's level of tolerance each time for approximately 2 cm/H2O. The primary goal was to eliminate respiratory distress, decrease the respiratory rate, and decrease the use of accessory respiratory muscles. The second goal was to improve blood gases and particularly PCO2 and pH. Necessary adjustments to NPPV settings were made based on clinical criteria and venous blood gases (VBGs). Supplemental oxygen, 3–15 L/min, was utilized to achieve peripheral oxygen saturation rate of ≥90% while the mask was fitted on the patient's face to minimize air leak. Cardiac monitoring and pulse oximetry as well as noninvasive blood pressure measurements were performed continuously during NPPV, and ventilator parameters (IPAP and EPAP) were recorded.

### 2.2. Outcome Measures

Epidemiologic data such as age and sex in addition to clinical and laboratory data including blood pressure, pulse rate, respiratory rate, accessory respiratory muscle use, and SaO_2_ and VBG parameters (HCO3, BE, PCO2, and pH) were measured at the time of enrollment and subsequently at 60 minutes and six and 24 hours after NPPV initiation. Support provided by the ventilator was quantified by measuring IPAP and EPAP, as well as total oxygen flow required to maintain peripheral oxygen saturation rate above 90%. Patient comfort while wearing the mask was assessed by two methods, namely, a visual analog scale (VAS) and a questionnaire. In use of a VAS, a score of 5 indicated that the patient was very comfortable while a score of 1 indicated extreme discomfort. Patients were asked about feeling pain in the forehead, nose, cheeks, and chin, air leak at eyes and mouth, dry nose and mouth, and compressive effects of mask on their faces. The patients' answers to the following questions were recorded in a questionnaire and each item was scored 0 to 3 in terms of intensity. The total score was calculated by adding the individual scores of each item mentioned above. Duration of NPPV use, endotracheal intubation rate, length of hospital stay, and in-hospital mortality rate were compared between the two groups as well.

### 2.3. Statistics

We used SPSS version 22 for statistical analysis. Data were analyzed using *t*-test and chi-square test considering 95% confidence interval, At *P* = 0.05 level of significance. Repeated measures ANOVA and Mann–Whitney *U* test were used to compare clinical and laboratory data between the two groups.

## 3. Results

The study was conducted between February and November 2014. There were no significant differences in age, sex, or disease severity (*P* > 0.05) in patients between the two groups. Patients in group one (13 men and 11 women) used TFM; 20 patients in this group (83.3%) had chronic obstructive pulmonary disease (COPD) exacerbations, while the rest had bronchiectasis, obstructive sleep apnea, and so forth. The mean age was 63.21 ± 10.22 years. Patients in group 2 (15 men and 9 women) used ONM. There were 20 patients with COPD exacerbation in this group, with a mean age of 67.83 ± 9.46 years. The demographics of the two groups are summarized in Table 1.

There was no difference in baseline heart rate of patients in the two groups. Similarly, there were no differences in respiratory rate, O_2_ saturation, or the use of accessory respiratory muscles at baseline between the two groups (Table 2).

At 6 hours, the patients in the TFM group had a significant reduction in their respiratory rate compared to those in the ONM group (*P* = 0.045).  Table 3 shows the recorded parameters at 1, 6, and 24 hours after initiation of NPPV in both groups.

As seen in Table 3, at six hours, PCO2 and HCO3 improved in patients using TFM, although there were no differences in VBG alterations between the two groups (*P* > 0.055).

With regard to patient comfort and acceptance, except for pain in the cheeks (*P* = 0.01), other parameters were similar between the two groups (Table 4).

However, the mean VAS score showed no difference in mask tolerance between the two groups (*P* = 0.25). Similarly, the total time of noninvasive ventilation (NIV) was similar in the two groups (*P* = 0.14) as shown in Table 5. Intubation was needed in two patients in ONM group and one patient in TFM group. One death occurred in each group.

Although both groups had similar IPAPs, EPAP was significantly higher in patients receiving NPPV via ONM (5.04 ± 0.81 cm H_2_O in TFM, 6.17 ± 1.74 in ONM) (*P* = 0.01, Table 6).

## 4. Discussion

The purpose of this study was to compare the efficacy and outcome of delivering NPPV to patients with acute respiratory failure using TFM or ONM. We hypothesized that TFM would be superior as it is reported to be more comfortable for patients. However, the mean VAS scores were similar in the two groups and different masks did not affect total NIV time, phobia, skin inflammation, or mortality rate.

Holanda et al. reported equal patient comfort with TFM and ONM, less air leak, nasal bridge pain, mouth and throat dry mucosa by TFM, and less claustrophobia with ONM [8]. Criner et al. also reported TFM to be superior to ONM with respect to air leak and patient comfort [6]. Chacur et al. reported better patient tolerance with TFM but, unlike our findings, they showed longer NIV time in TFM users [9]. Subsequently, a case series reported effective, safe, and acceptable experience in four patients, three having ALS (Amyotrophic Lateral Sclerosis) and nasal necrosis and one experiencing acute respiratory failure immediately after extubation [10]. Ozsancak et al. reported similar results to ours although they reported a higher success rate after three hours of NPPV with ONM versus TFM [11].

Concerning clinical and laboratory parameters, we found no obvious change or difference in heart rate or the use of accessory muscles after six hours of NIV, but respiratory rate was significantly lower in TFM users. This was consistent with the results of earlier studies, which showed no difference in heart rate, respiratory rate, or oxygen saturation after six hours of NPPV with TFM or ONM [8, 11]. On the contrary, Roy et al. reported significant reductions in heart rate and respiratory rate after one hour of NPPV via TFM in patients who had not tolerated other masks [7]. We compared VBG values (PCO_2_, pH, HCO_3_, and BE) at zero, one, six, and 24 hours after initiation of NPPV. We observed significant improvements in PCO_2_ and HCO_3_ after six hours in TFM group but the overall VBG alterations were the same in both groups. Criner et al. previously reported improved gas exchange in hypercapnic patients who received NPPV via TFM [6].

We also compared intubation rate and in-hospital mortality between the two groups, and similar to previous reports, we found no difference in either category [9, 11]. We also assessed patient comfort and found a lower score for pain in cheeks in patients receiving NPPV via TFM.

## 5. Conclusion

While TFM was more efficacious in reducing PCO2 and HCO3 during the first six hours of treatment, the blood gas alterations were the same in both groups based on repeated measures ANOVA once the patients were out of the acute phase of respiratory failure. Therefore, we may conclude that TFM was superior to ONM in acute phase of respiratory failure but not once the patients were out of acute phase. Our findings indicated that both TFM and ONM should be available in case of patient intolerance for one mask.

## Figures and Tables

**Table 1:** Age, gender, and comorbidities in patients in the two groups.

	TFM	ONM	*P* value
	*N* = 24	*N* = 24	
Age	63.21 ± 10.62	67.83 ± 9.46	0.118
Sex			
Male	13 (54.2%)	15 (62.5%)	0.558
Female	11 (45.8%)	9 (37.5%)	
Comorbidities			
ALS	1 (4.2%)	0 (0.0%)	>0.999
Bronchiectasis	2 (8.3%)	2 (8.3%)	
COPD	20 (83.3%)	20 (83.3%)	
ILD	0 (0%)	1 (4.2%)	
OSA	1 (4.2%)	1 (4.2%)	

ALS: Amyotrophic Lateral Sclerosis; COPD: chronic obstructive pulmonary disease; ILD: Interstitial Lung Diseases; OSA: obstructive sleep apnea.

**Table 2:** Comparison of clinical and oxygenation parameters at baseline (time zero) and six hours after initiation of treatment.

Efficacy	TFM	ONM	*P* value
	*N* = 24	*N* = 24	
	Clinical		
HR			
Time 0	91.12 ± 11.96	90.25 ± 11.87	>0.999^†^
Time 6 h	86.09 ± 6.47	86.13 ± 9.47	0.987
RR			
Time 0	21.50 ± 3.28	21.63 ± 1.86	0.175^†^
Time 6 h	19.30 ± 2.38	19.92 ± 1.93	0.045^†^
SPO2			
In room	84.17 ± 6.81	81.0 ± 6.65	0.063^†^
With O2	90.37 ± 4.55	92.33 ± 2.99	0.365^†^
Frequency of patients using accessory muscles			
Time 0	4 (16.7%)	10 (41.7%)	0.057
Time 6 h	1 (4.3%)	6 (25.0%)	0.055

^†^Mann–Whitney *U* test.

**Table 3:** Paraclinical parameters at one, six, and 24 hours after initiation of treatment.

Efficacy	TFM	ONM		*P* value
	*N* = 24	*N* = 24		
		Para-clinical		
		PH		0.249^††^
0	7.31 ± 0.05		7.30 ± 0.07	0.885^†^
1 h	7.34 ± 0.06		7.33 ± 0.07	0.881^†^
6 h	7.34 ± 0.05		7.33 ± 0.06	0.598
24 h	7.36 ± 0.05		7.34 ± 0.04	0.266

		PCO2		0.840^††^
0	85.41 ± 15.24		87.17 ± 18.68	0.723
1 h	72.11 ± 16.67		79.33 ± 16.22	0.139
6 h	70.11 ± 13.61		77.97 ± 11.83	0.040
24 h	70.39 ± 11.59		52.93 ± 30.19	0.137^†^

		HCO3		0.392^††^
0	42.11 ± 5.52		42.44 ± 6.76	0.852
1 h	39.71 ± 5.89		41.48 ± 8.00	0.523^†^
6 h	37.55 ± 5.05		41.81 ± 6.41	0.015
24 h	39.86 ± 4.92		38.78 ± 6.75	0.541

		BE		0.906^††^
0	11.29 ± 4.64		11.34 ± 6.06	0.977
1 h	12.23 ± 10.63		11.29 ± 6.85	0.932^†^
6 h	8.71 ± 3.62		11.45 ± 6.03	0.067
24 h	10.67 ± 4.19		9.36 ± 5.95	0.392

^†^Mann–Whitney *U* test; ^††^repeated measures ANOVA.

**Table 4:** Comfort of the masks based on the items evaluated in the questionnaire.

Severity	TFM	ONM	*P* value
	*N* = 24	*N* = 24	
Pain			
Forehead	0.26 ± 0.54^£^	0.33 ± 0.70	0.941^†^
Nasal bridge	0.35 ± 0.49	0.67 ± 0.70	0.113^†^
Cheeks	0.04 ± 0.21	0.50 ± 0.78	0.011^†^
Chin	0.26 ± 0.62	0.12 ± 0.45	0.359^†^
Air leakage			
Eye area	0.74 ± 1.10	0.50 ± 0.66	0.683^†^
Mouth	0.17 ± 0.39	0.33 ± 0.64	0.455^†^
Dry mucosa			
Throat	0.74 ± 0.81	1.04 ± 0.99	0.309^†^
Nose	0.61 ± 0.89	0.50 ± 0.72	0.808^†^
Skin inflammation			
Itching	0.13 ± 0.62	0.21 ± 0.59	0.356^†^
Burning	0 ± 0	0 ± 0	>0.999^†^
Other			
Compression	0.48 ± 0.51	0.50 ± 0.59	>0.999^†^
Phobia	0.13 ± 0.34	0.04 ± 0.20	0.281^†^
Total questionnaire Score	3.91 ± 2.41	4.75 ± 3.03	0.323^†^

^†^Mann–Whitney *U* test; £: mean ± standard deviation of score given by patients in each group to the mask (from 0 to 3) based on the intensity of each complication experienced.

**Table 5:** Acceptance score, NPPV total time, intubation rate, and mortality rate in the two groups.

	TFM	ONM	*P* value
	*N* = 24	*N* = 24	
Mean VAS score	3.13 ± 0.92	3.37 ± 0.92	0.255^†^
NPPV total time	6.52 ± 3.24	8.71 ± 4.75	0.141^†^
Intubation	1 (4.2%)	2 (8.7%)	0.609
Death	1 (4.2%)	1 (4.2%)	>0.999

^†^Mann–Whitney *U* test.

**Table 6:** IPAP, EPAP, and O2 requirement within the first hour in the two groups.

Ventilator setting	TFM	ONM	*P* value
	*N* = 24	*N* = 24	
IPAP (cm H_2_O)	13.21 ± 1.89	14.46 ± 3.13	0.273^†^
EPAP (cm H_2_O)	5.04 ± 0.81	6.17 ± 1.74	0.010^†^
O2 (Lit/min)	8.13 ± 4.87	9.92 ± 3.43	0.180^†^

IPAP: inspiratory positive airway pressure; EPAP: expiratory positive airway pressure.

^†^Mann–Whitney *U* test.

## Additional Points

*Study Limitations.* Six patients opted out after randomization due to previous unpleasant experiences with NPPV.
